# A predictive model for hyperuricemia among type 2 diabetes mellitus patients in Urumqi, China

**DOI:** 10.1186/s12889-023-16669-6

**Published:** 2023-09-07

**Authors:** Palizhati Abudureyimu, Yuesheng Pang, Lirun Huang, Qianqian Luo, Xiaozheng Zhang, Yifan Xu, Liang Jiang, Patamu Mohemaiti

**Affiliations:** 1 Medical Laboratory Center, First Affiliated Hospital of Xinjiang Medical University, No.137, Liyushan South Road, Xinshi District, Urumqi, 830001 China; 2 Xinjiang Uygur Autonomous Region, Xinjiang Medical University, No.567, North Shangde Road, Shuimogou District, Urumqi, 830017 China

**Keywords:** Type 2 diabetes Mellitus, Hyperuricemia, Risk factor, Prediction model, Nomogram

## Abstract

**Background:**

Patients with type 2 diabetes Mellitus (T2DM) are more likely to suffer from a higher uric acid level in blood—hyperuricemia (HUA). There are no conclusive studies done to predict HUA among T2DM patients. Therefore, this study aims to explore the risk factors of HUA among T2DM patients and finally suggest a model to help with its prediction.

**Method:**

In this retrospective research, all the date were collected between March 2017 and October 2019 in the Medical Laboratory Center of the First Affiliated Hospital of Xinjiang Medical University. The information included sociodemographic factors, blood routine index, thyroid function indicators and serum biochemical markers. The least absolute shrinkage and selection operator (LASSO) and multivariate binary logistic regression were performed to screen the risk factors of HUA among T2DM patients in blood tests, and the nomogram was used to perform and visualise the predictive model. The receiver operator characteristic (ROC) curve, internal validation, and clinical decision curve analysis (DCA) were applied to evaluate the prediction performance of the model.

**Results:**

We total collected the clinical date of 841 T2DM patients, whose age vary from 19-86. In this study, the overall prevalence of HUA in T2DM patients was 12.6%. According to the result of LASSO-logistic regression analysis, sex, ethnicity, serum albumin (ALB), serum cystatin C (CysC), serum inorganic phosphorus (IPHOS), alkaline phosphatase (ALP), serum bicarbonate (CO2) and high-density lipoprotein (HDLC) were included in the HUA risk prediction model. The nomogram confirmed that the prediction model fits well (χ^2^ = 5.4952, *P* = 0.704) and the calibration curve indicates the model had a good calibration. ROC analysis indicates that the predictive model shows the best discrimination ability (*AUC* = 0.827; 95% *CI*: 0.78–0.874) whose specificity is 0.885, and sensitivity is 0.602.

**Conclusion:**

Our study reveals that there were 8 variables that can be considered as independent risk factors for HUA among T2DM patients. In light of our findings, a predictive model was developed and clinical advice was given on its use.

## Background

Type 2 diabetes mellitus (T2DM) is a metabolic condition resulting from a combination of genetics, environmental factors, and dietary habits. The main symptoms of the condition are the inability to stabilise blood glucose levels and ineffective insulin secretion. The World Health Organization (WHO) has reported diabetes as the ninth leading cause of death in 2019 [1], with 1.5 million estimated deaths directly resulting from it, and the disease is likely to affect over 640 million adults by 2040 [2]. According to the International Diabetes Federation (IDF) report, the number of diabetes patients in China have been exceed 140 million currently [3]. Diabetes correlates with various diseases, such as thyroid dysfunction [4, 5], lipid metabolism disorder [6], purine metabolic disorder [7], cardiovascular disease [8], and cognitive function [9]. Long-term hyperglycemia leads to islet β-cells depletion and failure, ultimately resulting in hyperglycemia and metabolic decompensation [10].

Hyperuricemia (HUA) is a typical chronic metabolism illness brought about by purine metabolism disorder and serum uric acid (SUA) excretion disorder and it is a risk factor for diabetes, metabolic syndrome, obesity, high cholesterol, cardiovascular and kidney disease, [7, 11, 12]. According to a meta-analysis study [13], the prevalence of HUA was 16.4% in the Chinese population from 2000–2019. Epidemiological studies show that HUA is not only a key risk factor for gout, but also closely related to the diseases mentioned above as well as malignant tumors. As T2DM is a metabolic disorder, it often causes difficulty for patients to break down uric acid and leads to HUA, however the symptoms of HUA are not obvious, even more so among diabetic patients. There is a strong correlation between HUA and both diabetic peripheral neuropathy and diabetic microangiopathy [14]—resulting in nervous system and renal function damage, and increasing mortality among patients with diabetes.

T2DM as a risk factor for HUA has been poorly reported. There have been many studies on HUA risk prediction models, but most were developed on healthy populations. Eljaaly et al. [15] developed a logistic regression model that showed HUA was associated with hip circumference, total cholesterol, high-density lipoprotein, triglycerides, and serum creatinine. Huang et al. [16] constructed a nomogram prediction model for diabetic kidney disease. The predictive model was established to serve a clinical setting and so clear predictive ability, visualisation, and simple operation are necessary to ensure effective use. Thus, a clinically convenient and practical predictive model is imperative for early detection of HUA in patients with T2DM. In summation, the objective of our study was to investigate the potential risk factors associated with HUA in T2DM patients and develop an effective predictive model for clinical application.

## Methods

### Study population and definition

**Fig. 1:**
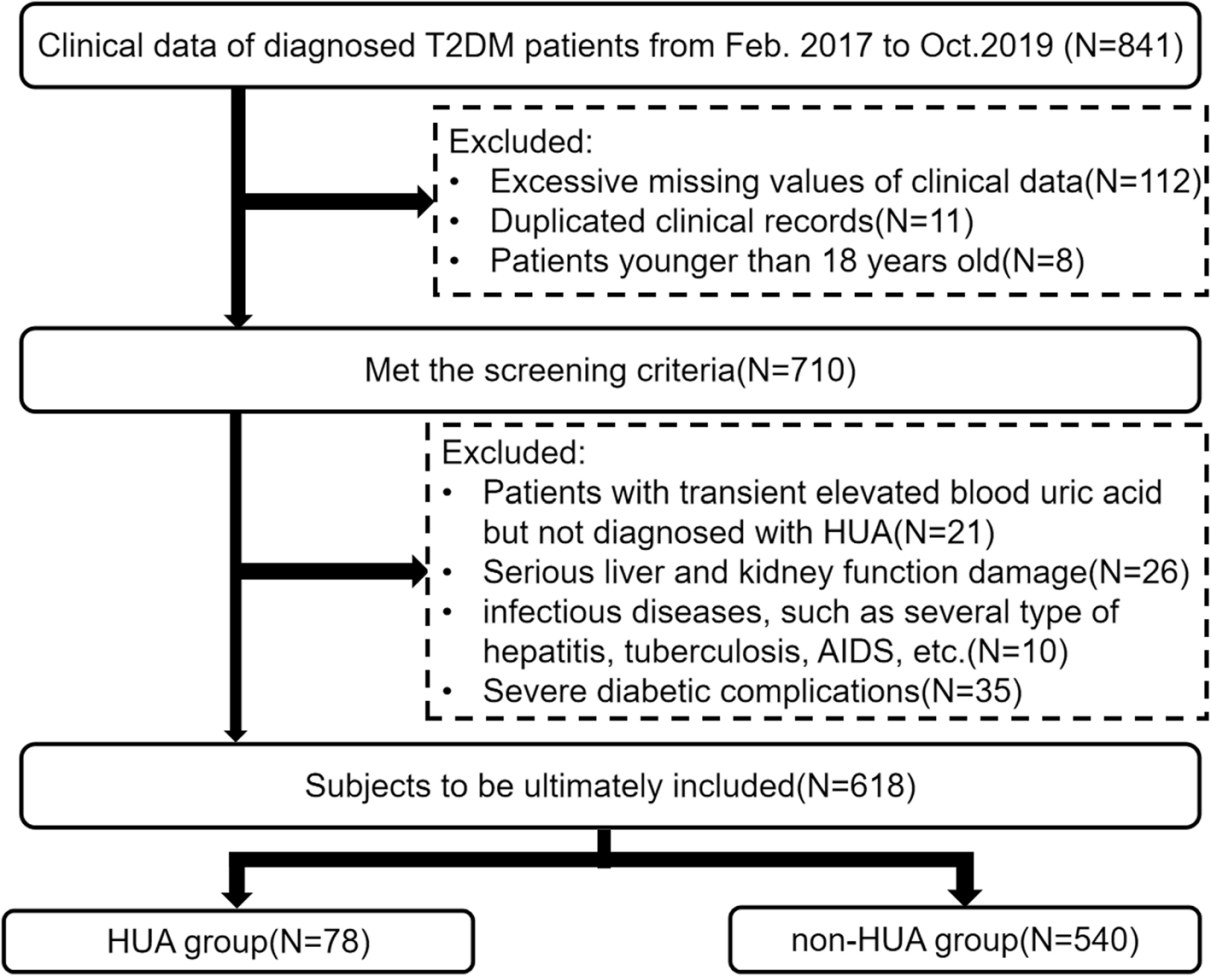
Flow chart of the research

In this retrospective study, we collected clinical data from the Medical Laboratory Center of the First Affiliated Hospital of Xinjiang Medical University, China, between March 2017 and October 2019. Patients diagnosed with T2DM – as defined by the Chinese Diabetes Society [17] – had typical symptoms, including excessive thirst and appetite, polyuria, and blood glucose in a specific range (i.e., greater or equal to 11.1 mmol/L, a fasting blood glucose of greater or equal to 7.0 mmol/L, an oral glucose tolerance test where the blood glucose after 2 h was greater or equal to 11.1 mmol/L, or a HbA1c of greater or equal to 6.5%). In total 841 patients followed the screening process shown in Fig. 1. Diagnosing HUA was based on sound diagnostic criteria (i.e. SUA of greater or equal to 420 mmol/L and 360 mmol/L for male and female respectively) [18–20]. All tests were performed whilst the patients were hospitalised. The study was approved by the ethics committee of the First Affiliated Hospital of Xinjiang Medical University (No.: K2303-11), and written informed consent was obtained from all participants.

The patients excluded were: (1) younger than 18 years old, (2) duplicated medical records, (3) effected by another type of diabetes, (4) not diagnosed with HUA but had transient elevated blood uric acid, (5) undergoing urate-lowering therapy or taking medications that affect blood uric acid levels, (6) suffering from a serious liver and kidney damage, (7) suffering from acute and chronic heart failure or heart failure; (8) suffering from infectious diseases, such as tuberculosis, AIDS, several type of hepatitis, etc., (9) suffering from severe diabetic complications.

According to the statistical analysis,the R package "epicalc" developed by Rodriguez [21] was used to calculate the sample size. The minimum sample size required was determined to be 169, considering the prevalence of HUA among T2DM patients in our research.

### Data collection

The baseline characteristics, collected from medical records, included the following results: (1) sociodemographic markers—age, gender and ethnicity, (2) blood glucose monitoring, (3) blood cell content tests, (4) thyroid function tests; (5) blood lipid tests; (6) renal function tests; (7) liver function indicators and (8) serum ionic concentration index.

### Statistical analysis

Statistical analysis and graph plotting were performed using SPSS 26.0 and R 4.1.3. Data are expressed as mean ± standard deviation, median (interquartile range, IQR) and numbers (percentage) for normal distribution quantitative variables, non-normal distribution quantitative variables and qualitative variables, respectively. The Student's t-test or the Wilcoxon rank-sum test was used to compare continuous data between two groups, while the chi-square test was used to compare categorical data between two groups. Given the strong collinearity among blood indicators in this study, the least absolute shrinkage and selection operator (LASSO) and multivariate logistic regression were used to screen characteristic variables. Then, we developed the nomogram according to the logistic regression. Receiver operating characteristic (ROC) curves and areas under the curve (AUC)—with 95% confidence intervals—were used to examine the predicting performance of the risk prediction model and each independent predictor for HUA. The decision curve analysis (DCA) was used to assess the maximum net benefit of the predictive model. The statistical difference was considered significant when the two-sided *P*-value was greater than 0.05.

## Results

### Baseline characteristics

A total of 618 eligible T2DM patients—410 males and 208 females—were included in this study, namely, 78 in the HUA group and 540 in the non-HUA group. In other words, the prevalence of hyperuricemia (HUA) among patients with type 2 diabetes is 12.6%. The mean ages of the non-HUA group and HUA group were (56.26 ± 12.17) and (57.46 ± 11.44) years, respectively. There were 17 (21.8%) well-controlled diabetic patients (i.e., with HbA1c less than 6.5%) in the non-HUA group, and 91 (16.7%) in the HUA group. The baseline characteristics of the participants were shown in Table 1. Participants with HUA were more likely to be female and different ethnic backgrounds contribute the different prevalence of HUA (*P* < 0.05). In addition, there were higher values of, TG, BUN, CREA, CysC, URIC, ALB, A/G, GGT and IPHOS in the HUA group compared to the non-HUA group (All *P* < 0.05), but lower values of HbA1c, GSP, MCH, APOA, HDLC, ALP, and CO2 (All *P* < 0.05). There was no significant difference in other indicators between HUA group and non-HUA group (*P* > 0.05).

**Table 1:** The comparison of general data and clinical indicators between HUA and non-HUA groups in T2DM patients

	All individuals(*N* = 618)	HUA group(*N* = 78)	non-HUA group(*N* = 540)	t/χ^2^/Z	*P*
**Sex (%)**					
male	410(66.3)	40(51.3)	370(68.5)	9.068	0.003^**^
female	208(33.7)	38(48.7)	170(31.5)		
**Ethnicity (%)**					
Han ethnicity	466(75.4)	47(60.3)	419(77.6)	11.045	0.001^**^
Ethnic minorities	152(24.6)	31(39.7)	121(22.4)		
Age, year	56.42 ± 12.075	57.46 ± 11.44	56.26 ± 12.17	-0.834	0.404
**Blood glucose index**					
GLU, mmol/L	7.91(5.96,110.09)	7.59(6.18,10.02)	7.98(5.89,11.23)	-1.003	0.316
≥ 11.1	151(24.4)	13(16.7)	138(25.6)	2.984	0.225
< 11.1	467(75.6)	65(83.3)	402(74.4)		
HbA1c, %	8.10(6.90,10.00)	7.65(6.58,9.20)	8.30(6.90,10.08)	-2.041	0.041^*^
≥ 6.5	511(82.7)	61(78.2)	450(83.3)	1.171	0.279
< 6.5	107(17.3)	17(21.8)	91(16.7)		
GSP, mmol/L	2.67(2.34,3.21)	2.53(2.28,2.94)	2.69(2.36,3.26)	-2.396	0.017^*^
**Blood cell content**					
EO%, %	2.20(1.40,3.20)	2.35(1.60,3.43)	2.10(1.40,3.10)	-0.754	0.451
HCT, %	42.80(39.60,45.20)	43.80(39.48,46.13)	42.70(39.60,45.10)	-1.365	0.172
LY%, %	33.07 ± 8.59	31.47 ± 8.36	33.30 ± 8.61	1.762	0.079
MCH, pg	30.10(29.10,31.20)	30.55(29.38,31.45)	33.25(27.33,39.58)	-2.280	0.023^*^
MCV, fl	90.70(87.80,93.40)	91.25(88.18,93.55)	90.70(87.73,93.40)	-0.793	0.428
NE%, %	56.80(50.78,62.50)	58.40(52.58,64.25)	56.65(50.50,62.18)	-1.611	0.107
PCT, %	0.24(0.20,0.27)	0.22(0.19,0.26)	0.24(0.20,0.27)	-1.884	0.06
PDW, %	16.28(14.90,17.33)	16.18(13.68,17.12)	16.28(14.98,17.37)	-0.856	0.392
PLT,10^9^/L	216.50(182.00,251.00)	203.50(174.00,237.00)	218.00(184.00,254.00)	-1.725	0.085
RBC,10^12^/L	4.72(4.35,5.02)	4.78(4.37,5.12)	4.71(4.35,5.00)	-0.862	0.389
RDW, %	12.90(12.50,13.40)	12.90(12.50,13.232)	12.90(12.40,13.40)	-0.121	0.904
WBC,10^9^/L	6.40(5.46,7.80)	6.74(5.70,8.06)	6.37(5.42,7.79)	-1.544	0.123
**Thyroid function index**					
FT3, pmol/L	4.47(4.09,4.91)	4.64(4.21,5.09)	4.45(4.08,4.88)	-1.721	0.085
FT4, pmol/L	15.86(14.25,17.62)	15.59(14.24,17.62)	15.89(14.25,17.63)	-0.461	0.645
**Blood lipid index**					
APOA, mmol/L	1.17(1.04,1.33)	1.12(0.99,1.26)	1.19(1.05,1.35)	-2.790	0.005^**^
APOB, g/L	0.92(0.74,1.11)	0.94(0.80,1.09)	0.92(0.73,1.12)	-0.715	0.475
CHOL, mmol/L	4.11(3.37,4.83)	4.22(3.58,4.87)	4.08(3.35,4.83)	-0.780	0.436
HDLC, mmol/L	1.06(0.86,1.27)	0.95(0.82,1.11)	1.08(0.87,1.30)	-3.558	< 0.001^**^
LDL-C, mmol/L	2.68(2.05,3.25)	2.69(2.02,3.18)	2.67(2.05,3.27)	-0.351	0.725
LP-A, mmol/L	112.25(55.96,206.18)	101.35(47.98,196.25)	114.50(57.91,210.88)	-1.408	0.159
TG, mmol/L	1.65(1.17,2.53)	2.22(1.59,3.59)	1.59(1.13,2.38)	-5.080	< 0.001^**^
**Renal function index**					
BUN, mmol/L	5.22(4.39,6.30)	5.90(4.48,7.03)	5.20(4.30,6.20)	-2.952	0.003^**^
CREA, μmol/L	66.00(54.53,77.16)	73.96(61.79,92.25)	65.00(53.88,76.00)	-4.914	< 0.001^**^
CysC, mg/L	0.74(0.61,0.92)	0.84(0.62,1.11)	0.73(0.60,0.90)	-2.662	0.008^**^
OSM, Osm/(kg.H_2_O)	293.24(288.93,296.95)	292.84(288.86,296.62)	293.31(288.97,297.06)	-0.098	0.922
URIC, mmol/L	303.00(246.00,362.00)	458.35(427.75,493.80)	291.59(238.63,340.29)	-13.476	< 0.001^**^
**Liver function index**					
ALB, g/L	41.50(38.80,44.20)	43.10(39.80,45.33)	41.30(38.70,44.00)	-2.864	0.004^**^
GLB, g/L	25.10(22.60,27.80)	25.00(21.80,26.63)	25.10(22.63,27.90)	-1.267	0.205
A/G	1.671 ± 0.35	1.76 ± 0.33	1.66 ± 0.35	-2.499	0.013^*^
ALP, U/L	73.00(61.00,88.55)	68.20(54.50,82.25)	74.00(61.00,90.875)	-2.512	0.012^*^
ALT, U/L	19.13(13.90,29.00)	20.10(14.58,35.43)	18.95(13.70,28.50)	-1.439	0.150
AST, U/L	17.20(14.00,21.65)	18.50(15.00,24.18)	17.10(13.93,21.00)	-1.899	0.058
AST/ALT	0.86(0.69,1.10)	0.85(0.66,1.06)	0.86(0.69,1.11)	-0.848	0.058
DBIL, μmol/L	3.48(2.50,4.70)	3.10(2.32,4.49)	3.50(2.50,4.70)	-1.215	0.224
GGT, U/L	25.00(17.00,37.7)	28.05(19.78,47.00)	24.60(17.00,37.00)	-2.187	0.029^*^
T-BIL, μmol/L	10.20(7.40,14.00)	10.25(7.10,15.30)	10.20(7.43,13.80)	-0.659	0.510
TBA, μmol/L	3.87(2.50,6.08)	4.03(2.52,6.50)	3.82(2.49,6.02)	-0.836	0.403
IBIL, μmol/L	6.68(4.40,9.73)	7.99(3.80,12.53)	6.60(4.48,9.48)	-1.243	0.214
**Serum ionic concentration**					
CA, μmol/L	2.26(2.18,2.33)	2.28(2.18,2.34)	2.26(2.18,2.32)	-0.914	0.361
CL, μmol/L	103.00(101.00,105.33)	103.00(100.75,105.40)	103.00(101.00,105.28)	-0.037	0.970
K, mmol/L	3.91(3.69,3.25)	3.94(3.76,4.22)	3.90(3.69,4.13)	-1.232	0.218
NA, mmol/L	141.10(139.00,143.00)	140.30(138.86,142.70)	141.10(139.00,143.00)	-0.964	0.335
MG, mmol/L	0.85(0.79,0.89)	0.84(0.77,0.89)	0.85(0.80,0.89)	-1.380	0.168
IPHOS, mmol/L	1.18(1.06,1.30)	1.25(1.14,1.38)	1.17(1.05,1.29)	-3.579	< 0.001^**^
CO2, mmol/L	24.86 ± 2.74	23.92 ± 2.81	24.99 ± 2.70	3.263	0.001^**^

Abbreviations: *HUA* Hyperuricemia, *GLU* Random blood glucose, *HbA1c*, Glycosylated hemoglobin A1c, *GSP*, Glycosylated serum protein, *EO* Percentage of eosinophils, *HCT* Hematocrit, *LY*% The percentage of lympholeukocyte, *MCH* Mean cellular haemoglobin contents, *MCV* Mean corpuscular volume, *NE*% Neutrophil percentage, *PCT* Thrombocytocrit, *PDW* Platelet distribution width, *PLT* Platelet count, *RBC* Red blood cell count, *RDW* Red blood cell distribution width, *WBC* White blood cell count, *FT3* Free triiodothyronine, *FT4* Free thyroxine, *APOA* Apolipoprotein A, *APOB* Apolipoprotein B, *HDLC* High-density lipoprotein, *LDL-C* Low density lipoprotein, *LP-A* Lipoprotein A, *TG* Triglyceride, *BUN* Blood Urea Nitrogen, *CREA* Blood creatinine, *CysC* Serum cystatin C, *OSM* Urine osmolality, *URIC* Blood uric acid, *ALB* Serum albumin, *GLB* Serum globulin, *A/G* Serum albumin/ globulin ratio, *ALP* Alkaline phosphatase, *ALT* Glutamic-pyruvic transaminase, *AST* Glutamic oxaloacetic transaminase, *DBIL* Direct bilirubin, *GGT* Glutamyltransferase, *T-BIL* Total serum bilirubin, *TBA* Total bile acid, *IBIL* Indirect bilirubin, *CA* Serum calcium, *CL* Serum chlorine, *K* Serum Kalium, *NA* Serum sodium, *MG* Serum magnesium, *IPHOS* Serum inorganic phosphorus, *CO2* Serum bicarbonate

Data are shown as mean ± standard deviation, median (interquartile range), or frequency (percentage). *P < 0.05, **P < 0.01

### Screening of HUA-related risk factors

**Fig. 2:**
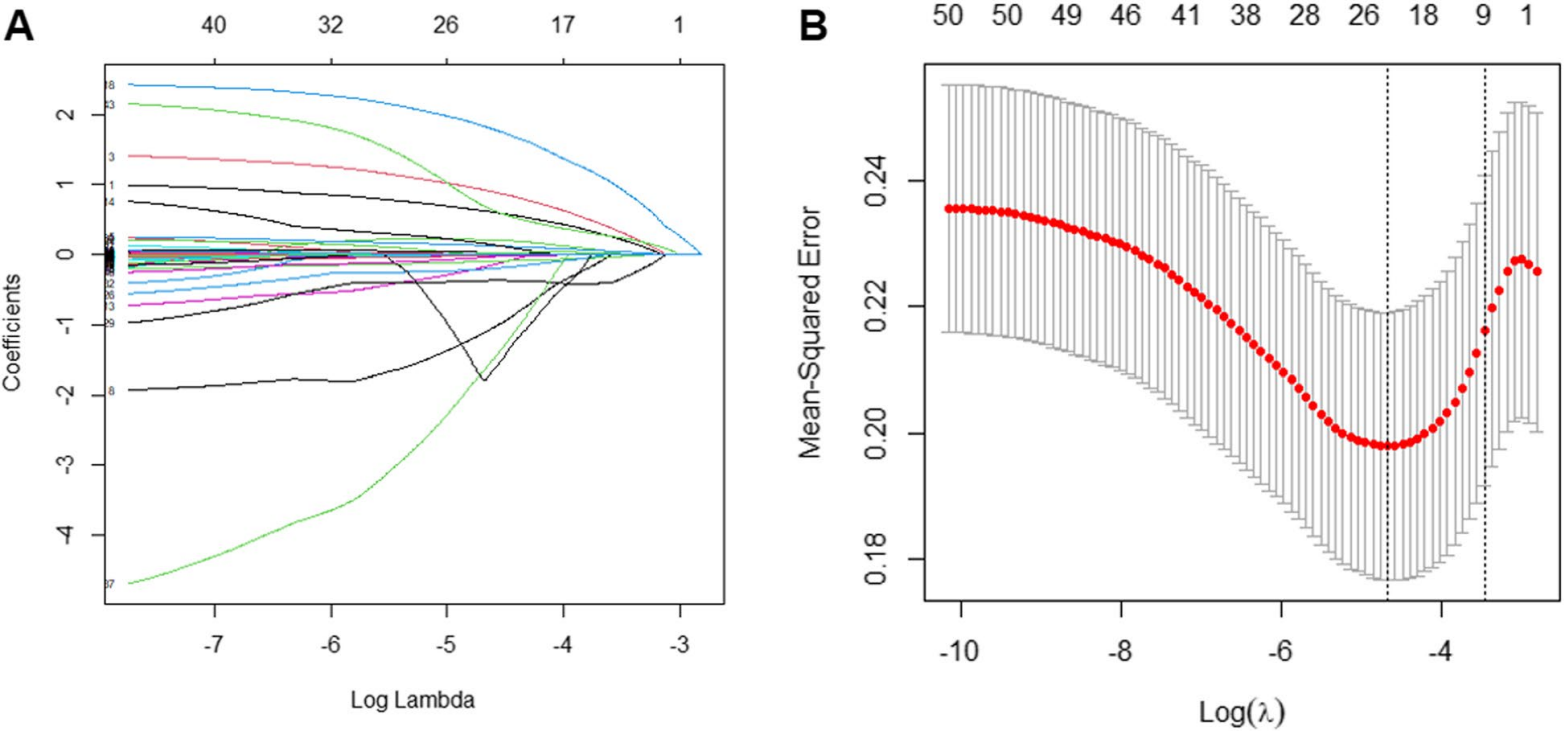
Characteristic variables were screened using LASSO regression analysis. **A** LASSO coefficient profiles of the nine characteristic variables. A coefficient profile plot was produced against the log (lambda) sequence. **B** The selection of the best parameter (lambda) in the LASSO model uses tenfold cross-validation. The relationship curve between partial likelihood deviation (binomial deviation) and log (lambda) was plotted. Dotted vertical lines were drawn at the optimal values by using the minimum criteria and the one SE of the minimum criteria (the one SE criteria). Abbreviations: *LASSO* Least absolute shrinkage and selection operator, *SE* Standard error

As shown in Fig. 2, the variables were screened by LASSO regression and tenfold cross-validation, the λ was taken when the model error was the one standard error of the minimum (the one SE criteria), and nine indicators were finally screened, including sex, ethnicity, ALB, CysC, IPHOS, ALP, CO2, HDLC and TG. Then, these variables were incorporated into multivariate logistic regression analyses shown in Table 2. Sex, ethnicity, ALB, CysC, IPHOS, ALP, CO2 and HDLC were identified as independent risk factors for HUA in the T2DM patients.

**Table 2:** Binary logistic regression analysis of the risk factors for HUA in T2DM patients

	Reference	B	OR (95% CI)	S.E	*P*
Constant	-	-6.914	0.001	2.434	0.005^**^
Sex	Male	0.926	2.525(1.421–4.485)	0.293	0.002^**^
Ethnicity	Han ethnicity	1.237	3.445(1.918–6.188)	0.299	< 0.001^**^
ALB	-	0.191	1.211(1.121–1.308)	0.039	< 0.001^**^
IPHOS	-	1.638	5.146(1.098–24.125)	0.788	0.038^*^
HDLC	-	-1.659	0.190(0.068–0.529)	0.522	0.001^**^
CO2	-	-0.187	0.829(0.747–0.921)	0.053	< 0.001^**^
CysC	-	2.566	13.011(5.229–32.374)	0.465	< 0.001^**^
ALP	-	-0.022	0.978(0.965–0.991)	0.007	0.001^**^

Abbreviations: *OR* Odds Ratio, *S.E.* Standard error, *ALB* Serum albumin, *CysC* Serum cystatin C, *IPHOS* Serum inorganic phosphorus, *ALP* Alkaline phosphatase, *HDLC* High-density lipoprotein, *CO2* Serum bicarbonate

^*^P < 0.05, **P < 0.01

### The build and analysis of HUA risk prediction model

**Fig. 3:**
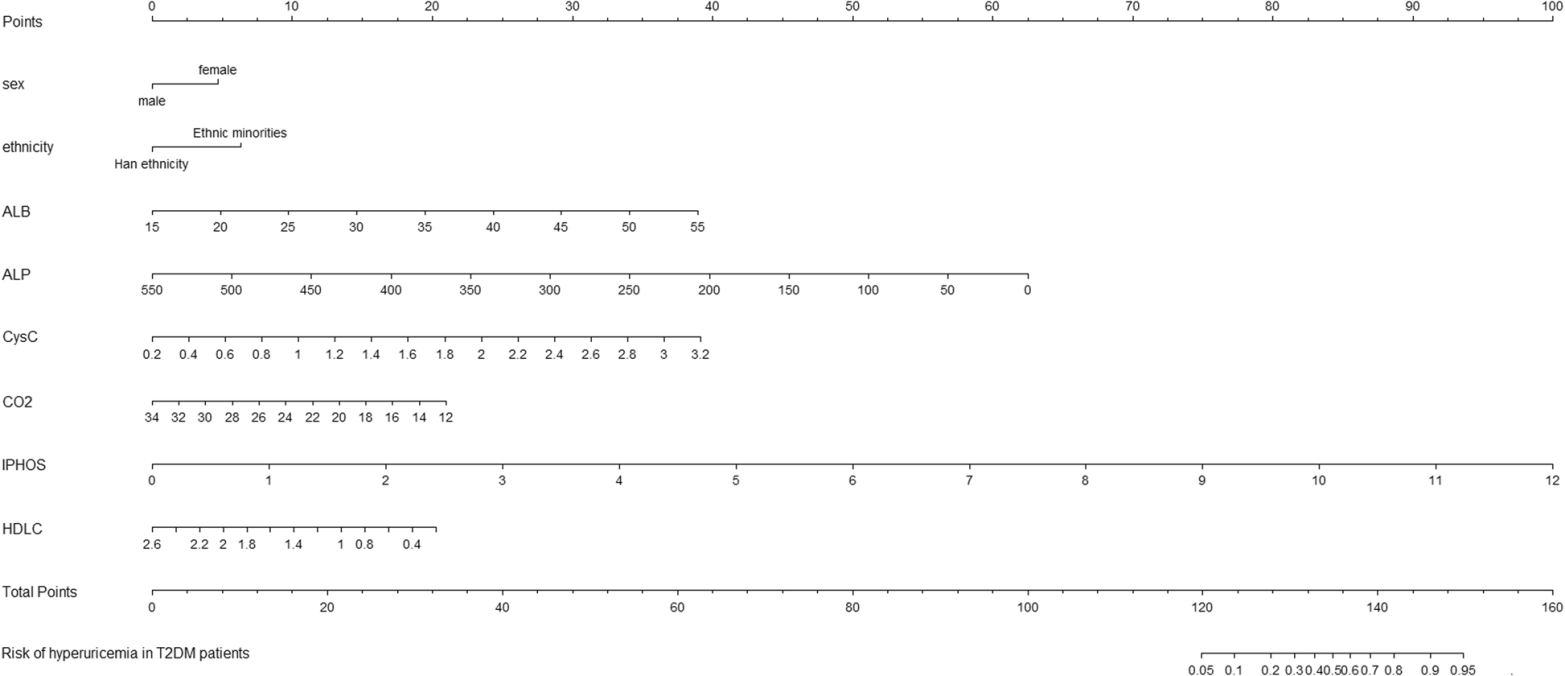
Nomogram for prediction HUA among T2DM patients. Abbreviations: *HUA* Hyperuricemia, *ALB* Serum albumin, *CysC* Serum cystatin C, *IPHOS* Serum inorganic phosphorus, *ALP* Alkaline phosphatase, *HDLC* High-density lipoprotein, *CO2* Serum bicarbonate

**Fig. 4:**
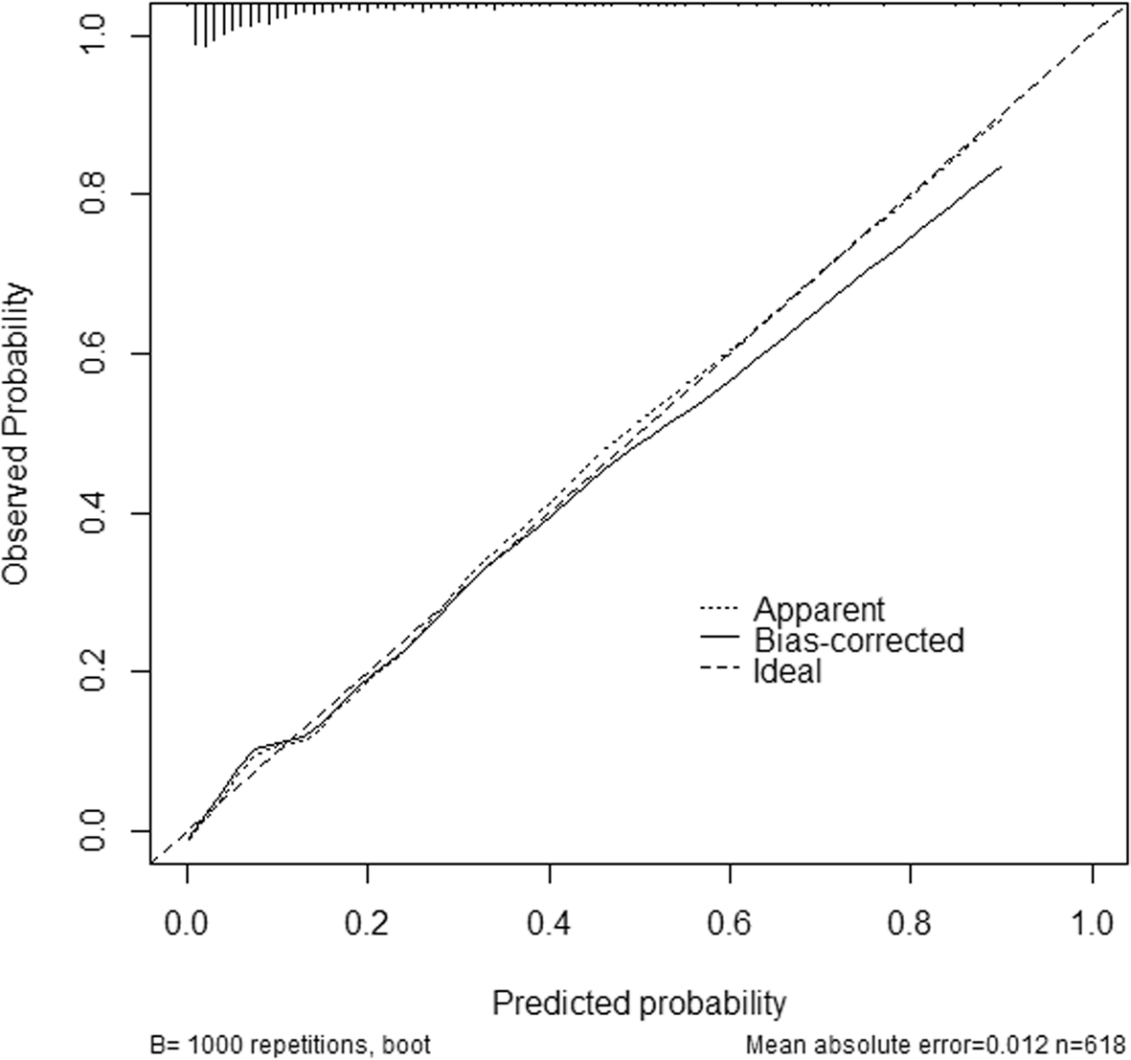
The calibration plot for nomogram

Based on the outcomes of LASSO-logistic regression results, the nomogram was created and shown in Fig. 3. The sum of the corresponding scores of each variable (including sex, ethnicity, ALB, CysC, IPHOS, ALP, CO2 and HDLC) was the individual risk score, which gave an estimated probability of HUA risk in T2DM patients. The Bootstrap method was used to test and verify the nomogram model giving a measure of internal validation, and the calibration curve (see Fig. 4) was drawn after the raw data was sampled 1,000 times. The Hosmer–Lemeshow goodness-of-fit test showed that the prediction model fits well (χ^2^ = 5.4952 and *P* = 0.704), which mean that there is no statistically significant difference between the risk prediction value and the actual observation value. The results showed that the model accurately predicts the risk of HUA in T2DM patients.

### The evaluation of the HUA risk prediction model in T2DM patients

**Fig. 5:**
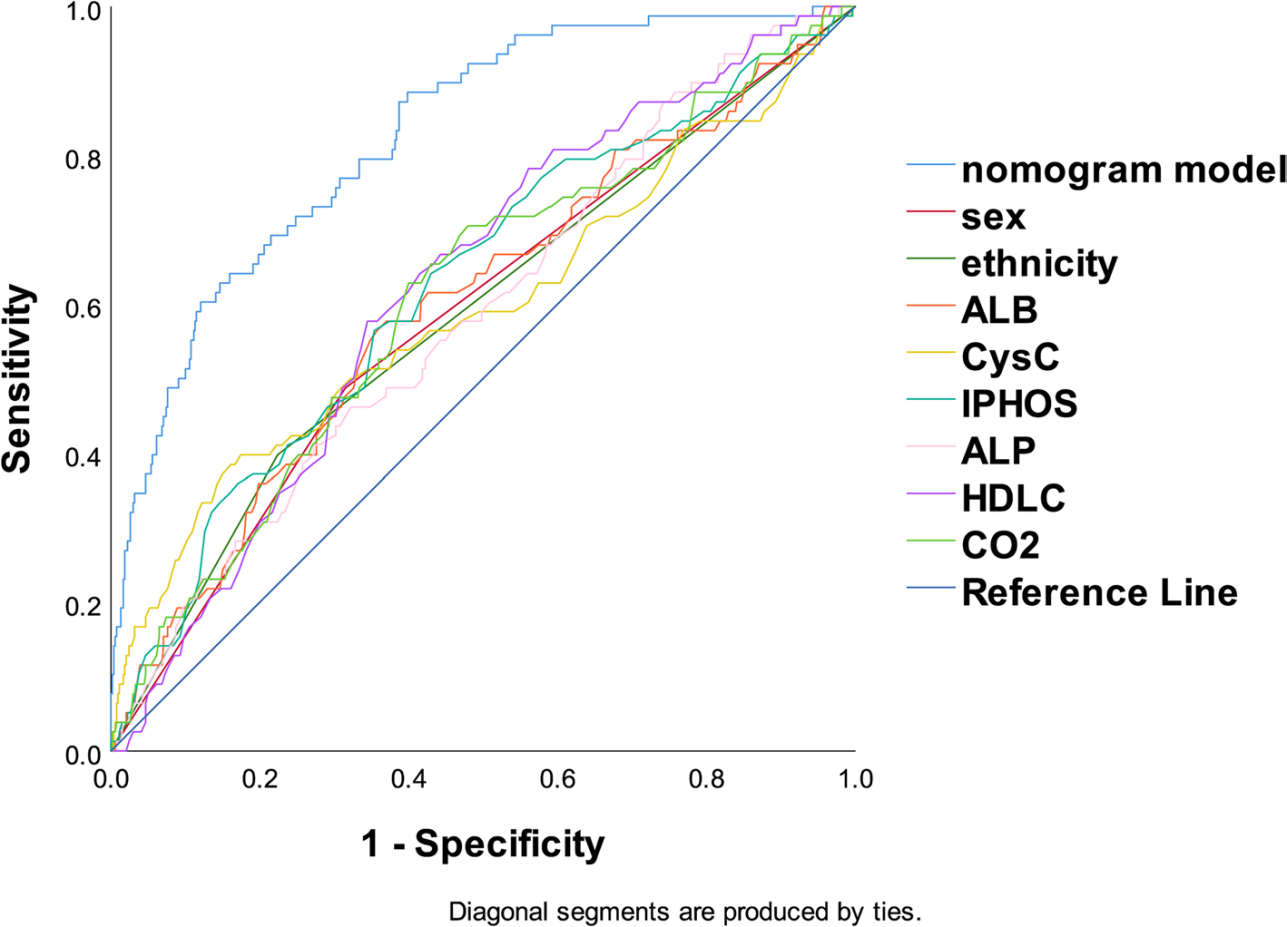
The ROC curve of the risk factor for predicting HUA in T2DM patients. Abbreviations: *ROC* Receiver operating characteristic, *HUA* Hyperuricemia, *ALB* Serum albumin, *CysC* Serum cystatin C, *IPHOS* Serum inorganic phosphorus, *ALP* Alkaline phosphatase, *HDLC* High-density lipoprotein, *CO2* Serum bicarbonate

**Fig. 6:**
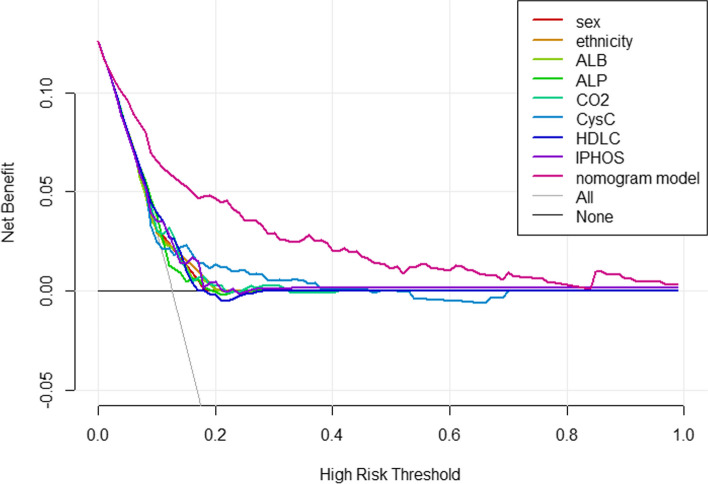
The DCA curve of the predictive model. Abbreviations: *DCA* Decision curve analysis, *ALB* Serum albumin, *CysC* Serum cystatin C, *IPHOS* Serum inorganic phosphorus, *ALP* Alkaline phosphatase, *HDLC* High-density lipoprotein, *CO2* Serum bicarbonate

The ROC curves for identifying T2DM participants with HUA was shown in Table 3 and Fig. 5. IPHOS showed the best discrimination ability (*AUC* = 0.625; 95% CI: 0.557–0.693) among all 8 indicators (Delong's test all *P* < 0.05), including sex (*AUC* = 0.586), ethnicity (*AUC* = 0.87), ALB (*AUC* = 0.6), CysC (*AUC* = 0.593), IPHOS (*AUC* = 0.625), ALP (*AUC* = 0.588), HDLC (*AUC* = 0.625) and CO2 (*AUC* = 0.61). The AUC for the combination of the above 8 parameters, which gave a prediction for HUA among T2DM patients, is 0.827 (95% *CI*: 0.78–0.874, *P* < 0.001), specificity is 0.885, and sensitivity is 0.602. Collectively, its predictive performance was better than individual factors. Based on the result of DCA, the predictive model was also outperformed in comparison to individual indicators, see Fig. 6.

**Table 3:** The ROC curve of the risk factor for predicting hyperuricemia in T2DM patients

	AUC	AUC 95%* CI*		S.E	*P*	Youden Index	Cut-off	Specificity	Sensitivity
		Lower	upper						
Sex	0.586	0.517	0.655	0.035	0.014^*^	0.172	0.500	0.685	0.487
Ethnicity	0.587	0.516	0.658	0.036	0.013^*^	0.173	0.500	0.776	0.397
ALB	0.600	0.531	0.670	0.035	0.004^**^	0.207	42.850	0.643	0.564
CysC	0.593	0.517	0.669	0.039	0.008^**^	0.224	0.995	0.839	0.385
IPHOS	0.625	0.557	0.693	0.035	< 0.001^**^	0.211	1.205	0.570	0.641
ALP	0.588	0.522	0.654	0.034	0.012^*^	0.139	62.500	0.678	0.462
HDLC	0.625	0.562	0.687	0.032	< 0.001^**^	0.232	0.965	0.656	0.577
CO2	0.610	0.542	0.678	0.035	0.002^**^	0.228	2.421	0.600	0.628
Prediction model	0.827	0.780	0.874	0.024	< 0.001^**^			0.885	0.602

Abbreviations: *ALB* Serum albumin, *CysC* Serum cystatin C, *IPHOS* Serum inorganic phosphorus, *ALP* Alkaline phosphatase, *HDLC* High-density lipoprotein, *CO2* Serum bicarbonate

^*^P < 0.05, **P < 0.01

## Discussion

Our result indicated the prevalence of HUA among patients with T2DM, identified independent risk factors associated with HUA, and established a HUA-related prediction model for T2DM patients in Urumqi city, China. The prevalence of HUA in our study population was 12.6%, which is lower than the reported prevalence among patients with diabetes (32%) [22]. To comprehensively assess the independent risk factors for HUA among T2DM patients, we conducted a thorough screening using demographic information, medical history, and laboratory test results from a cohort of 618 T2DM patients. Utilizing LASSO-logistic regression analysis, we identified several independent risk factors associated with HUA among T2DM patients, including sex, ethnicity, CysC, ALB, IPHOS, ALP, CO2, and HDLC.

Previous studies have verified estrogens have a uricosuric effect on the kidney to excrete SUA [23], which leads to the difference in the prevalence of HUA between males and females. Our research findings demonstrate that females with T2DM are more susceptible to HUA than their male counterparts, which aligned with the conclusions drawn in the study conducted by Eljaaly et al. [15]. Meanwhile, according to the reports and our result, it was indicated that different ethnic backgrounds ( e.g., ApoE E4, IL-8, IL-18 gene polymorphism) may contribute the different prevalence of HUA [24].

It is worthy to notice that serum cystatin C (CysC), serum albumin (ALB) and blood inorganic phosphorus (IPHOS) were significant risk factors for HUA among T2DM patients in blood indicators. CysC is an endogenous protease inhibitor in the cystatin superfamily, it contributes to intracellular protein breakdown [25]. Serum CysC plays a crucial role in reflecting renal function and SUA levels [26]. ALB is a significant human protein, maintains osmotic pressure, pH, and aids fatty acid transport [27]. Our study disclosed high serum ALB as a risk factor of HUA in T2DM patients. Chronic inflammation has a close relation with the concentrate of ALB [28]. T2DM patients exhibit an ALB increase, this possibly due to chronic inflammation [29]. To serum IPHOS, a cross-sectional study indicated that there is no substantial link between SUA and serum calcium/phosphorus levels [30]. While, in patients with primary hyperparathyroidism, a positive association between SUA and serum calcium/serum IPHOS concentration has been suggested [31]. Variability in serum IPHOS concentration, may influenced by a high phosphorus diet factors [32]. In our research findings, the impact of serum IPHOS and serum CysC on HUA is significant enough to warrant attention from clinical practitioners. Therefore, for T2DM patients, these two indicators can serve as important clinical monitoring parameters to prevent the occurrence of HUA and renal impairment.

In the current multivariate logistic regression analysis, high-density lipoprotein (HDLC), serum bicarbonate (CO2) and alkaline phosphatase (ALP) were emerged as protective factors for HUA in T2DM patients, and essential components for our prediction model. Prior studies have highlighted the independent predictive role of lower HDLC levels in HUA development [33, 34]. Lower HDLC levels to increased susceptibility to kidney impairment, thereby reducing uric acid excretion [35]. CO2 is a crucial factor in regulating body fluid acid–base and electrolyte balance, and low level of CO2 reflects impaired renal function to a certain extent [36]. ALP, involved in phosphorylation and cellular metabolism, correlates with asymptomatic HUA [29], implying roles in cell signaling, lipid metabolism, and uric acid modulation. Overall, we incorporate the above 8 indicators related to HUA into nomogram model.

A nomogram is a visual graph using distinct lines to predict clinical events. Our model's validity, discrimination, and application were verified. ROC analysis shows improved disease prediction (AUC = 0.827) and specificity (88.5%) vs. individual indicators. Goodness-of-fit and calibration plot confirm model accuracy. Huang et al. [16] reported BMI, HbA1c, eGFR, hyperlipidemia as DKD risk factors (AUC = 0.843). This nomogram for HUA influencing factors also showed clinical applicability. These similar studies highlight of nomograms in disease prediction. Visual models aid early HUA diagnosis and prevention in T2DM, which is crucial for resource-limited areas.

## Limitations

The study developed a predictive model using 8 blood indicators, and the resulting nomogram showed promising predictive performance. However, the study has limitations. Firstly, due to its retrospective single-center design, there's potential for selection bias, which might affect the prediction model and experimental results with regional traits. Secondly, the patient sample size with HUA was insufficient for meaningful analysis. Thirdly, the study doesn't establish a causal relationship between influencing factors and HUA in T2DM patients. Thus, future research should involve prospective cohort or case–control studies to confirm our assumptions. Additionally, not considering anthropometric indicators in the analyses limits the scope of findings. Consequently, the conclusions drawn from our study warrant cautious interpretation.

## Conclusion

This study supplements the evidence for the ability of each indicator to identify HUA among T2DM patients and provides theoretical support for early screening of diabetes complicated by HUA in the T2DM population. Our results have demonstrated that the proposed model achieved a higher value of AUC compared to previously models, indicating promising potential for early identification and diagnosis of HUA in T2DM patients. Regular monitoring of the relevant biomarkers included in the model can help reduce the incidence of HUA, thus mitigating the onset of associated comorbidities and improving the quality of life in diabetic patients.

## Data Availability

The database generated during the current study is not publicly available due to privacy restrictions. The data used to support the results of this study can be obtained from the corresponding authors as required. Please send specific suggestions for future cooperation to the corresponding authors.
